# Predatory marketing and false health promotion on social media: risk pathways in diet, fitness, and supplement communication

**DOI:** 10.3389/fpubh.2026.1709812

**Published:** 2026-02-23

**Authors:** Youjing Huang, Xinchen Leng, Zirong Tian

**Affiliations:** 1 College of Educational Science, Bohai University, Jinzhou, Liaoning, China; 2 College of Social Sciences and Humanities, Northeastern University, Seattle, DC, United States; 3 Media Communication College, Dankook University, Yongin-si, Republic of Korea

**Keywords:** false beliefs, health misinformation, predatory health marketing, psychological susceptibility, social media

## Abstract

As the commercial circulation of health content on social media continues to intensify, large volumes of fitness, nutrition, and wellness information lacking scientific grounding are repeatedly pushed to users, heightening the likelihood that psychologically susceptible individuals internalize distorted beliefs and engage in harmful practices. This study examines the mechanism through which exposure to such content influences psychological vulnerability, strengthens false health beliefs, shapes risky behavioral choices, and ultimately affects perceived health status. Using 482 valid survey responses, we conducted confirmatory factor analysis, structural equation modeling, bootstrap mediation tests, multigroup comparisons, and robustness checks to investigate these pathways. The findings show that exposure significantly increases psychological vulnerability, which further promotes endorsement of inaccurate beliefs and encourages risky health behaviors, leading to poorer health outcomes. All indirect effects were statistically supported, and the moderating influences of Government Support and avoidance tendencies revealed that individual and contextual factors can alter the strength of the mechanism. Robustness analyses demonstrated that the belief variable is indispensable, as removing it led to substantial declines in model fit, indicating that adverse outcomes arise not from isolated exposure but from the gradual reinforcement and internalization of misleading claims. These results clarify the psychological and behavioral processes through which misleading health information exerts its influence in digital environments and provide empirical grounding for regulatory strategies that seek to intervene in the formation and consolidation of erroneous health beliefs rather than relying solely on limiting content visibility.

## 1 Introduction

In the global digital health communication landscape, social media has transformed public access to diet, fitness, and wellness information, accelerating dissemination while simultaneously intensifying misinformation risks. Commercialized domains such as supplements, body-sculpting regimes, and extreme fitness practices increasingly rely on influencer-mediated content disguised as personal experience rather than advertising, often exaggerating benefits or obscuring ingredients and safety information. Evidence across regions confirms this trend: Potvin Kent et al. (1) show that adolescent dietary perceptions are shaped through sensory appeal, social endorsement, and concealed promotion; Ricke and Seifert (2) reveal that nearly 70 % of supplements marketed by German health influencers contained inaccuracies or misleading claims; and Yeung et al. (3) document widespread medical-style rhetoric without verified sourcing on TikTok. Such patterns not only erode informational reliability but also heighten health and body-image anxieties, fueling harmful intentions such as the use of appearance-enhancing drugs, as observed in U.S. adults by Ganson et al. (4). In a similar vein, Dennehy et al. (5) show that women’s fitness-related social media use often blends aspirations for health with repeated exposure to risky exercise routines and restrictive dieting practices, suggesting that gendered platform cultures can normalize high-risk behaviors under the appearance of empowerment. Correspondingly, early scholarship on false health information focused on describing message features and exposure patterns but offered limited psychological explanation. Subsequent work expanded toward cognitive-affective mechanisms, emphasizing how algorithmic amplification and perceived social authority shape belief formation and vulnerability in participatory media environments (6, 7). Research grounded in psychological models such as the Health Belief Model and social comparison theory demonstrates how exposure to idealized bodies and wellness narratives heightens perceived susceptibility and internalized pressures (8, 9), while meta-evidence indicates media literacy interventions are less effective in highly aestheticized and fragmented environments (10). Ziapour et al. (11) further show that short-video platforms reduce users’ ability to verify authenticity due to cognitive masking effects produced by visual stimulation and influencer trust. However, psychological susceptibility—long noted as a driver of misbelief adoption in contexts of anxiety and self-concept fragility (12)—has often been treated as a control variable rather than a central explanatory construct. Moreover, despite emerging attempts to link media literacy and health literacy to behavior (13), studies rarely integrate emotional, cognitive, and algorithmic dynamics into a coherent process model. This fragmented development has advanced foundational insights yet still overlooks how social media functions as an affect-driven meaning-construction space where repeated emotional activation, heuristic processing, and commercial persuasion jointly shape vulnerability.

Building on these observations, although previous research has extensively documented the prevalence and complex drivers of false health information on social media, current scholarship has not yet established a coherent explanatory chain that traces the full process from exposure to mistaken belief and ultimately to harmful health behavior (14). Much of the existing literature continues to emphasize message features and platform-based interventions, including algorithmic warning labels, misinformation flags, and account restrictions, but offers limited insight into how individuals cognitively interpret misleading content, allocate psychological resources, and make health-related decisions in environments shaped by algorithmic recommendations and information overload (15, 16). Torun et al. (17) similarly demonstrate that differences in e-health literacy shape how individuals apply the Health Belief Model in real-world health decisions, reinforcing the need to treat literacy as a dynamic moderator rather than a static attribute. In fast-paced digital contexts, individuals must evaluate and respond to information continuously, yet this internal reasoning process remains insufficiently theorized in health communication research (18). Moreover, platform regulations frequently function as symbolic signals rather than effective safeguards among populations with lower levels of media and scientific literacy, resulting in what Rodrigues et al. (19) describe as an “illusion of governance.” At the same time, susceptibility has been identified as a central psychological mechanism in misinformation acceptance and a critical entry point for intervention design (20), yet prior models rarely treat it as a core variable. To address these gaps, this study establishes an integrated framework that links social media exposure, cognitive appraisal, psychological vulnerability, and subsequent health decisions, while also examining the moderating roles of perceptions of platform governance and Information Avoidance behaviors. On the basis of this framework, the study formulates four research questions (RQs) that correspond to its core pathways and variable design:

RQ1: How do the frequency and content features of exposure to false health information on social media affect individuals’ subjective risk perceptions?
RQ2: Do higher levels of media literacy and scientific literacy enhance individuals’ ability to recognize false information?
RQ3: Does risk susceptibility mediate the relationship between exposure to false information and subsequent health behavior responses?
RQ4: Does sustained exposure to false information generate measurable health consequences, such as misinformed health beliefs, delayed care-seeking, or deviations in health behaviors?

Anchored in the four research questions that structure the analytical design and reflect the key dimensions of exposure, cognition, susceptibility, governance perception, and health-related behavior, this study advances existing work by shifting attention from fragmented message-level or platform-centered explanations toward the cognitive and emotional mechanisms through which misleading health content shapes individual judgment and decision-making. Drawing on the Health Belief Model, Social Comparison Theory, and Media Literacy Theory, the framework foregrounds psychological susceptibility and illusory belief formation as central explanatory elements, responding to concerns that prior models have not adequately captured micro-level belief construction in algorithmically driven, high-velocity media environments (18) and often understate the role of recipient-side vulnerabilities (19). By incorporating perceptions of platform governance and Information Avoidance as contextual moderators, the model clarifies how institutional safeguards and individual defensive strategies jointly influence the transition from exposure to belief and from belief to health behavior. In light of the empirical findings, this study defines predatory health content as digitally mediated health-related communication that strategically obscures its commercial intent, exaggerates benefits or downplays risks, and is designed primarily to steer users’ perceptions and behaviors in favor of promotional rather than health interests. Conceptually, this study reconceptualizes predatory health content as a structured persuasive tactic designed to shape perception and conduct, rather than a simple informational artifact. Practically, it offers a pathway-based perspective for risk classification in high-exposure domains such as supplement promotion and extreme fitness culture, emphasizes cognitive empowerment to cultivate information resilience and strengthen individual resistance to misleading content (20), and highlights the importance of embedding media-scientific literacy training in everyday digital environments for younger users. By centering psychological mediation, contextual moderation, and empirically validated behavioral pathways, this work provides a grounded basis for platform governance, public health education, and policy refinement in contemporary media settings characterized by accelerated information flow and heightened emotional persuasion.

## 2 Literature review

This chapter reviews research on digital health misinformation and predatory health marketing and organizes it into five connected parts. The first part looks at how platform design and commercial incentives shape the spread of predatory health information and clarifies how this study defines the problem. The second part summarizes empirical work on media exposure, psychological vulnerability, and the formation of false health beliefs, with a focus on how user level susceptibilities interact with platform features. The third part follows these beliefs forward to examine how they guide risky health behaviors and contribute to negative health outcomes. The fourth part turns to platform governance and user moderation, asking whether current responses genuinely protect vulnerable users or leave important risks unmanaged. The final part brings these strands together, identifies gaps that remain in the literature, and introduces the conceptual model that underpins the empirical analysis.

### 2.1 The digital dissemination features and problem definition of predatory health information

In contemporary digital health communication, social media platforms have reshaped how wellness information is encountered, interpreted, and internalized, embedding algorithmic ranking, aesthetic persuasion, and participatory feedback into everyday meaning-making practices. Unlike traditional media environments governed by editorial oversight, visibility on social platforms is largely driven by interaction metrics and emotional resonance, creating fertile conditions for persuasive yet scientifically unverified content to thrive (21). Narrative-oriented analyses further show that misleading health content spreads through dialogic pathways that blend personal storytelling with pseudo-expert authority, producing emotionally charged narratives that heighten credibility and intensify persuasive force within these algorithmically curated environments (22). Recent studies show that predatory health information strategically leverages sensory appeal, pseudo-scientific language, and influencer-mediated credibility to alter user perception and stimulate consumption: Potvin Kent et al. (1) find that adolescents’ dietary beliefs are shaped through concealed endorsements and narrative persuasion, Ricke and Seifert (2) report that most supplement promotions in Germany contain misleading claims, and Yeung et al. (3) document widespread use of medical rhetoric without credible sourcing on TikTok. Exposure to such content correlates with heightened behavioral intentions, as evidenced by Ganson et al. (4) in the context of appearance-enhancing drugs. Algorithmic feeds, immersive interfaces, and short-form video formats intensify cognitive activation and emotional arousal, reinforcing internalized pressures related to body ideals and health performance (6, 8). Yet conventional media-literacy interventions frequently weaken within visually saturated and socially endorsed environments (10, 11), indicating that learning to “recognize misinformation” is insufficient when content presentation is designed to bypass analytical processing. Empirical work further shows that anxiety, identity fragility, and appearance concerns magnify psychological susceptibility, heightening belief adoption and behavioral imitation (7, 12), although these factors remain under-addressed as primary explanatory variables in many models. Commercial design intensifies these pathways by linking exposure, stimulation, and purchase cues through in-feed commerce, gamified cues, and social validation signals (23). Meanwhile, dominant platform responses continue to rely on post-hoc deletion of content rather than adjusting ranking architectures that systematically privilege provocative signals, creating what Rodrigues et al. (19) describe as a governance illusion. Vulnerable groups—including youth, individuals managing chronic conditions, and women—face disproportionate emotional and cognitive burdens under these conditions (24, 25). Taken together, these insights reveal that predatory health information functions not as isolated false content but as a structurally embedded persuasive system aligned with commercial incentives and psychological vulnerabilities, requiring explanatory approaches that integrate cognitive-emotional processes, interface design, and platform power structures (13, 26).

### 2.2 Media exposure, susceptibility, and the formation of false beliefs

In contemporary digital health communication, social media platforms have reshaped how wellness information is accessed, interpreted, and absorbed, folding algorithmic ranking, visual persuasion, and interactive feedback into everyday meaning-making practices. Unlike legacy media environments guided by editorial control, social platforms prioritize interaction signals and emotional salience, creating fertile conditions for persuasive but weakly verified health narratives to circulate at scale (21). Empirical studies reveal that predatory health content routinely blends sensory appeal, pseudo-scientific language, and influencer authority to alter risk perception and stimulate consumption. Potvin Kent et al. (1) show that adolescents’ dietary beliefs are shaped through concealed endorsements and narrative persuasion; Ricke and Seifert (2) report that a majority of supplement promotions in Germany contain misleading or exaggerated claims; Yeung et al. (3) document the pervasive use of medical phrasing without credible sourcing on TikTok; and Ganson et al. (4) find that exposure to body-sculpting content increases intentions to use appearance-enhancing substances. These patterns are intensified by algorithmic feeds and immersive short-video formats, which heighten cognitive activation and emotional arousal, reinforcing internalized ideals of bodily perfection and self-optimization (6, 8). Traditional media-literacy interventions often lose effectiveness in such visually saturated and socially endorsed environments (10, 11), suggesting that the ability to identify misinformation alone is insufficient in ecosystems designed to bypass deliberative reasoning. Psychological susceptibility, fueled by anxiety, identity fragility, and body-image concerns, increases acceptance of misleading claims and imitation of risky conduct (7, 12), yet remains insufficiently foregrounded as a primary explanatory factor in many analytical models. Commercial design choices further accelerate these processes by linking exposure, emotional stimulation, and purchasing impulses within seamless in-platform commerce and feedback loops (23). Meanwhile, platform moderation tends to function reactively, removing content after circulation while leaving intact the ranking architectures that privilege provocative and affective cues, resulting in what Rodrigues et al. (19) describe as a governance illusion. Vulnerable populations such as adolescents, women, and individuals managing chronic conditions therefore face disproportionate exposure and psychological strain (24, 25). As these dynamics accumulate, predatory health content emerges not as isolated falsehoods but as a structurally embedded persuasion system supported by commercial incentives, platform recommendation logic, and user-level vulnerabilities. This recognition underscores the need for explanatory approaches that examine cognitive and emotional pathways together with interface design and platform governance structures (13, 26).

### 2.3 How false beliefs drive risky health behaviors

Recent evidence also indicates that health conspiracy theories function as catalysts for rigid belief structures and distrust in medical authorities, reinforcing pathways through which misinformation translates into adverse behavioral choices (27). Once false health beliefs solidify, their influence over behavior becomes persistent and directive, sustained by motivational commitment and socially reinforced imitation rather than by simple information acquisition. Individuals in digital environments are not passive recipients but actively construct belief frameworks that feel self-determined, enabling harmful decisions to appear rational and evidence based from the believer’s own perspective. Empirical research demonstrates that conspiratorial and alternative health beliefs strongly predict vaccine refusal, resistance to public health guidance, and reliance on unverified remedies, with belief-driven motivation acting as the dominant explanatory force (28). During the pandemic, declining trust in institutions allowed many individuals to follow internally constructed logics rather than medical advice, illustrating how false beliefs gain authority through the social ecologies in which they circulate (29). Social media environments further accelerate this process by granting interactive visibility to misleading claims, normalizing risky practices, and creating a sense of collective endorsement that encourages imitation even when misinformation originates from non-expert users (30). Emotional language and repeated circulation reinforce this dynamic, allowing assertions such as the claim that vitamins can replace vaccines to accrue credibility through repetition rather than empirical validation. At the same time, entrenched belief formation reflects not only repeated exposure and emotional reinforcement but also the structural phenomenon of data voids, whereby high user demand intersects with limited credible information to allow misleading narratives to dominate information spaces (31). Recent computational research further shows that data voids are not static but can be actively exploited or reshaped through algorithmic or community-driven processes, as illustrated by studies on political data voids and AI-supported interventions (32). These findings suggest that information scarcity is not merely a background condition but an evolving structural feature of digital environments, one that interacts dynamically with exposure and emotional reinforcement to accelerate the consolidation of false beliefs. In such contexts, individuals seeking health guidance encounter content ecologies skewed by informational scarcity and algorithmic ranking, intensifying susceptibility and strengthening belief certainty. These epistemic distortions mean that harmful choices often arise not from ignorance but from misplaced certainty and a conviction that personal intuition or bodily “self-knowledge” outweighs expert consensus (33). Attempts to correct misinformation under these conditions can provoke defensive reasoning and identity reinforcement, trapping individuals in rigid cognitive loops and reducing openness to future correction (30). At the collective level, false beliefs weaken compliance with preventive measures, undermine institutional legitimacy, and destabilize public health governance (34), while also producing psychological consequences such as anxiety, shame, cognitive fatigue, and decision paralysis among those caught in ongoing informational conflict. Ultimately, the progression from mediated exposure to entrenched belief and embodied practice reflects the interconnected roles of personal motivation, social imitation, information-availability asymmetries, and recursive cognition, highlighting the need for interventions that act early in the belief-formation process and address the informational and emotional infrastructures through which predatory narratives become socially embedded and behaviorally consequential. In this communication environment, misleading health content does not emerge as an occasional deviation but circulates through coordinated aesthetic cues, commercial incentives, and algorithmic amplification. Rather than functioning as isolated inaccuracies, such narratives operate as a structured persuasion system built on emotional arousal, pseudo-scientific authority, and consumption-driven design. To show how these mechanisms unfold across platforms and cultural settings, Table 1 presents representative cases from major social media environments, demonstrating narrative strategies, targeted user groups, and associated behavioral risks. These cases indicate that predatory health messages are neither platform-neutral nor randomly distributed; instead, they disproportionately concentrate on populations marked by heightened anxiety or informational vulnerability, manipulate trust asymmetries, and trigger avoidant or alternative health behaviors that gradually erode evidence-based decision-making and public health capacity.

**Table 1:** Representative cases of health misinformation campaigns on social media.

Case	Platform and topic	Misinformation narrative	Target group(s)	Observed/expected risk behavior
1	Facebook: COVID-19 vaccine	“mRNA vaccines alter human DNA”	General public; vaccine-hesitant adults	Vaccine refusal; decline in compliance with immunization guidelines
2	Instagram: fitness supplements	“Herbal fat-burning pills are safe and free of side effects”	Young women; body-image-anxious users	Use of unregulated supplements; insomnia; cardiovascular strain
3	TikTok: natural therapies	“Vitamin C or herbal tea can replace vaccines and antibiotics”	Adolescents; chronic patients	Substitution of medical treatment; delayed professional care
4	YouTube: anti-aging products	“Detox drinks cleanse the body and prevent aging”	Middle-aged women; wellness seekers	Overconsumption of detox products; electrolyte imbalance; distrust in medical advice
5	Weibo: conspiracy narratives	“Hospitals hide the truth; pharmaceutical companies profit from illness”	Chinese netizens; conspiracy-vulnerable groups	Rejection of hospital care; reliance on folk remedies; erosion of trust in public health institutions

Source: author’s contribution.

### 2.4 Platform governance and user moderation: protection or neglect

As digital health communication becomes increasingly intricate, social media platforms have assumed the role of digital gatekeepers, shaping users’ exposure to information and influencing risk perception through both technical architecture and governance protocols. Although these mechanisms are nominally designed to curb misinformation, their actual effects are ambivalent, raising the question of whether they genuinely protect vulnerable publics or merely institutionalize selective attention and structural neglect. Contemporary platforms primarily rely on forms of soft governance, including fact-checking labels, warning notices, and collaborative annotation systems. Evidence from Drolsbach et al. (35) in PNAS Nexus shows that Twitter’s Community Notes improved user trust in platform verification and that its transparency and decentralized oversight reduced resistance to moderation, suggesting that legitimacy is grounded less in the sophistication of detection systems and more in perceived procedural fairness. Yet this “moderate control” model is not without limitation. Gomes and Sultan (36) in the Harm Reduction Journal argue that platform responses are uneven, with intervention more likely when political and economic stakes are low and less likely when misinformation overlaps with sensitive commercial or institutional interests. Such patterns reinforce entrenched power dynamics and blur the line between platforms as neutral infrastructures and as moral adjudicators of public knowledge. Crucially, the effectiveness of these systems rests not only on technical accuracy but on user interpretation. Experimental research by Horne and Nevo (37) indicates that users often comply with warning cues even when skeptical of their validity, a behavior characterized as ecological rationality, driven by pragmatic adaptation rather than genuine conviction. Although this compliance may suppress harmful actions in the short term, it risks cultivating passive reliance on platform cues and reducing autonomous judgment over time. Conversely, when governance is perceived as partial or punitive, users may exit into alternative information spaces or engage in defensive silence and identity-based resistance. Zhang et al. (38) demonstrate that among vaccine-hesitant populations, warning labels sometimes intensified attachment to narratives framed as censored, driving misinformation into more opaque circulation channels. These findings suggest that platform governance operates within a fragile equilibrium: excessive intervention provokes backlash and politicization, while insufficient regulation allows predatory content to proliferate unchecked. Rather than a linear regime of technological authority imposed on passive audiences, governance unfolds within dynamic psychosocial feedback loops shaped by trust, identity, and perceived fairness. Strengthening institutional transparency, embedding meaningful user participation, and recognizing governance as a cognitive as well as regulatory process will be essential to mitigating misinformation risks without eroding public trust or further stratifying information access.

### 2.5 An integrative perspective and response to theoretical gaps

Although prior research has explored the production, circulation, and regulation of health misinformation across communication, sociology, and psychology domains, most studies examine discrete stages or single determinants, such as emotional arousal, perceived credibility, or the effects of fact-checking labels on belief formation (39). While these works have generated valuable insights into specific mechanisms within digital ecosystems, they often remain fragmented, lacking an integrated account of how psychological reactions unfold over time and translate into differentiated behavioral trajectories in complex information environments (40). To address this theoretical fragmentation, the present study introduces a structured model linking platform governance, risk perception, cognitive appraisal, and behavioral responses, while incorporating moderating factors including platform trust, media literacy, and self-efficacy. By doing so, it responds to calls for multi-layered frameworks that capture how governance measures, such as warning labels, community annotation systems, and filtering interventions, shape user judgments through cognitive and emotional pathways rather than exclusively through immediate compliance effects (41). In contrast with research that treats governance primarily as a direct determinant of trust or misinformation acceptance, this approach highlights the indirect, psychologically mediated role of governance in shaping shifts between avoidance, engagement, and rejection of information. A further gap in existing studies lies in the treatment of users as largely passive actors, with insufficient recognition of active strategies such as selective ignoring, withdrawal from interaction, or resistance to perceived over-intervention (42). By integrating these behavioral repertoires into its moderating structure and empirically testing the conditioning roles of media literacy and platform trust, this study extends governance evaluation research beyond one-directional models and identifies heterogeneity as an essential analytical dimension. Current interdisciplinary debates increasingly challenge the adequacy of platform-centered explanations and emphasize the need to embed user psychology within broader socio-technical models (39). Consistent with this trajectory, the present framework accounts for platform architecture, cognitive processing, and behavioral adaptation simultaneously, addressing critiques that user cognition is often undertheorized. At the same time, empirical studies reveal that while governance mechanisms can heighten vigilance and risk perception, excessive intervention generates information fatigue, desensitization, or defensive avoidance, producing counterproductive effects (40, 42). Building from these observations, the study advances the governance-overreach hypothesis, arguing that when interventions exceed perceived legitimacy thresholds, users shift toward self-protective strategies that diminish or even reverse regulatory effectiveness. By explicitly theorizing this process and grounding it in emerging evidence, the study contributes a refined and context-sensitive model of digital health governance that reflects the interplay between platform signals, cognitive evaluation, and adaptive behavior.

## 3 Methods

This chapter explains how the study was designed and implemented in order to examine the mechanisms through which health misinformation takes effect. The first part outlines the overall research design and theoretical framework, describing how the Health Belief Model, Social Comparison Theory, and Media Literacy Theory are linked into a single analytical sequence and how the structural equation model is specified. The second part introduces the sampling strategy, the characteristics of the participants, and the data collection procedures on the Wenjuanxing platform, together with the main quality control measures. The third part presents descriptive statistics for the key variables to provide an empirical backdrop for the modeling work. The final part sets out the indicator framework and measurement strategy, explaining how each construct is operationalized and how the statistical tools in R are used to test reliability, validity, and structural relationships.

### 3.1 Research design and theoretical framework

**Figure 1:**
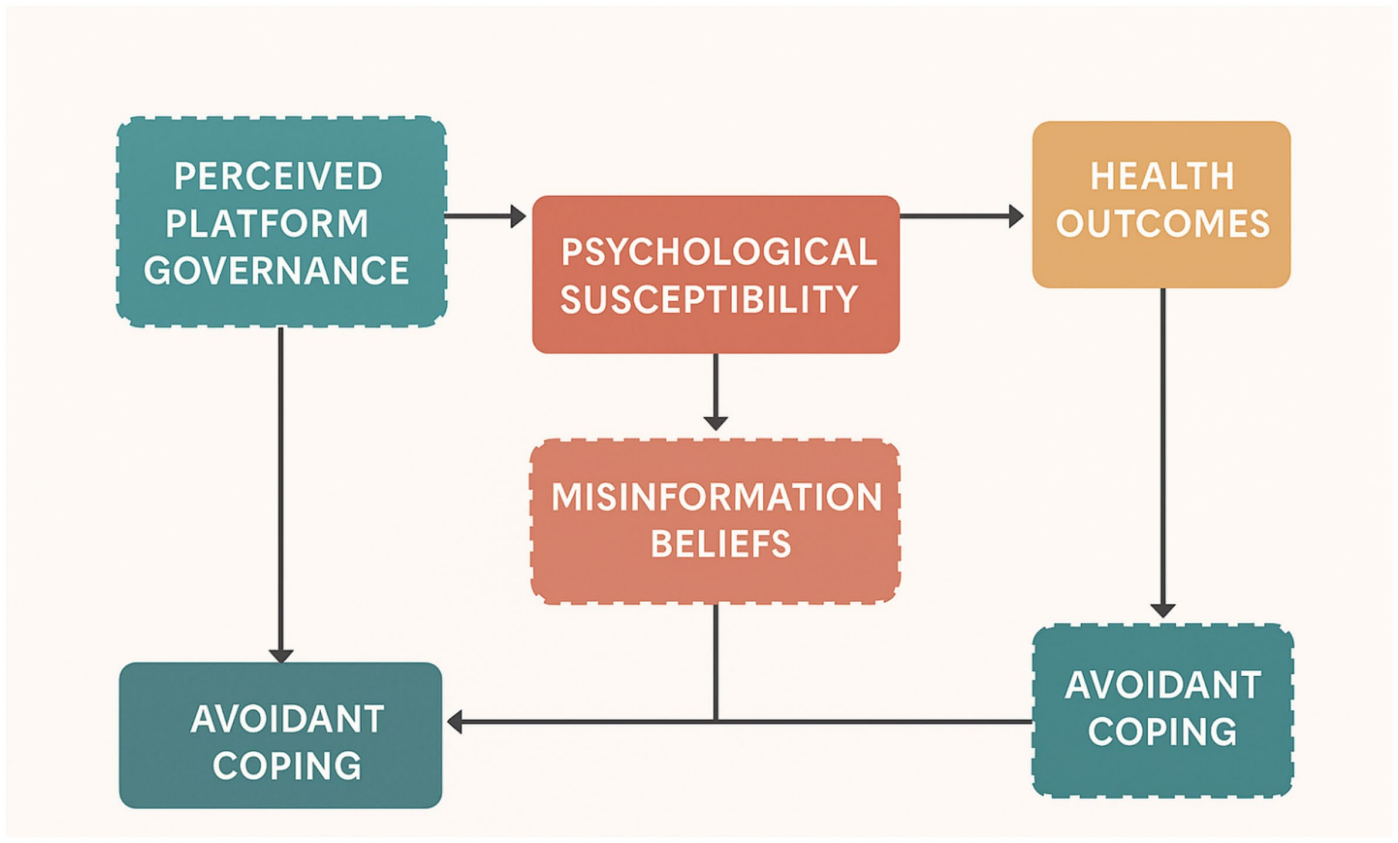
Conceptual framework of health misinformation effects. Source: author’s contribution.

SEM is a multivariate technique used to estimate relationships among latent constructs and observed indicators within a single integrated model (e.g., (43)). Building on this approach, the present study employed a cross-sectional quantitative research design and applied Structural Equation Modeling (SEM) to examine how exposure to predatory health content on social media influences individuals’ psychological susceptibility, the formation of false health beliefs, engagement in risky health behaviors, and subsequent health outcomes. The theoretical model integrates the Health Belief Model, Social Comparison Theory, and Media Literacy Theory to construct a sequential pathway that captures how exposure triggers psychological vulnerability, how such vulnerability shapes cognitive interpretations, and how distorted beliefs are translated into behavioral decisions and health consequences. To address contextual and individual variations, two moderating constructs—Government Support and information avoidance—were incorporated to capture the role of institutional trust and defensive coping strategies in shaping the strength of the pathway. All constructs were measured using adapted versions of internationally validated scales, and their corresponding items and sources are summarized in Table 2. This framework provides a theoretically grounded and empirically testable structure for understanding how misleading health information exerts its influence within contemporary digital environments (Figure 1).

**Table 2:** Measurement constructs, sample scale items.

Construct	Scale items (examples)	Scale sources or conceptual inspiration	Adaptation notes
Social media exposure to health content	“I frequently encounter content about fitness, nutrition, or supplements on social media.”	Frison and Eggermont (52), Social Media Exposure Scale	Items adapted to reflect Chinese digital platforms (e.g., Douyin, Xiaohongshu)
Psychological susceptibility/health anxiety	“I feel anxious about my health when viewing health-related posts online.”	Whiteley Index-7 (53)	Selected psychological vulnerability items and contextualized for digital health content
Misinformation belief (false health beliefs)	“Natural remedies or influencer advice can be more reliable than medical professionals.”	Misinformation susceptibility scale (54)	Adapted wording to reflect supplement/fitness misinformation narratives
Risky health behaviors	“I have tried health products/diets recommended by influencers without consulting professionals.”	CAM Usage Indicators (adapted from Harris et al. (55))	Added behaviors relevant to supplements, detox, fitness challenges
Health outcomes	“Overall, I feel my recent physical health has been good/poor.”	Scale source: Cella et al. (56)	Used global health items to assess perceived health status
Government support	“I believe social platforms can effectively identify and limit misleading health information.”	Scale source: adapted from Kelton et al. (57)	Reworded to fit Chinese social media environment
Information avoidance	“When health information online makes me uncomfortable, I tend to skip or scroll past it.”	Information avoidance scale (58)	Modified to platform interaction context (scrolling, swiping, muting reminders)

Sources, and adaptation notes.

### 3.2 Participants and data collection

Data were collected in June 2025 using the Wenjuanxing online survey platform,^1^ one of the most widely used online survey tools in China, to distribute the questionnaire and obtain responses efficiently across diverse user groups. A stratified sampling strategy was adopted based on gender, age (18–45 years), educational level, and frequency of social media use in order to capture user groups most likely to encounter health-related content online. Prior to formal data collection, a pilot test with 30 participants was conducted to assess clarity, linguistic comprehensibility, logical flow, and completion time, and adjustments were made accordingly. Eligibility criteria required participants to have used social media at least three times per week over the past 3 months, to have recently encountered content related to fitness, nutrition, body management, or dietary supplements, and not to be employed in medical, public health, or nutrition-related professions. Data quality was ensured through attention-check items, IP duplication checks, minimum completion time thresholds, and logical consistency screening.

A total of 482 valid responses were retained. Women accounted for 50.6% of the sample, with a mean age of 31.6 years (SD = 8.3). Most participants (82.8%) held a bachelor’s degree or above, and 43.2% were frequent or constant social media users, aligning with characteristics of populations most exposed to health-related content in digital environments. All data processing and analyses were conducted in R (4.3.0) on macOS. The tidyverse package was used for data cleaning and coding, psych for reliability testing, and lavaan for confirmatory factor analysis, structural model estimation, mediation testing, moderation analysis, and multi-group comparisons. Data preparation procedures included handling reverse-coded items, evaluating missing data, examining skewness and kurtosis, and assessing model fit, ensuring that the analytic process was rigorous, transparent, and replicable.

### 3.3 Descriptive statistics

To provide a clear overview of the sample characteristics and establish the empirical context for the subsequent analyses, Table 3 presents the descriptive statistics of all study variables separately for female and male participants.

**Table 3:** Descriptive statistics of study variables by gender.

Variable	Female n	Female mean ± SD	Female median [IQR]	Female range	Male n	Male mean ± SD	Male median [IQR]	Male range
Age (years)	244	32.00 ± 8.25	32.00 [25.00, 39.00]	18–45	238	31.29 ± 8.27	31.00 [24.00, 39.00]	18–45
SM use frequency	244	3.14 ± 1.40	3.00 [2.00, 4.00]	1–5	238	3.02 ± 1.46	3.00 [2.00, 4.00]	1–5
Risk exposure	244	3.07 ± 1.48	3.00 [2.00, 4.00]	1–5	238	3.09 ± 1.40	3.00 [2.00, 4.00]	1–5
Psychological vulnerability	244	4.20 ± 0.89	4.00 [4.00, 5.00]	2–5	238	4.20 ± 0.87	4.00 [4.00, 5.00]	2–5
Health belief	244	4.69 ± 0.57	5.00 [4.00, 5.00]	3–5	238	4.74 ± 0.47	5.00 [5.00, 5.00]	3–5
Risky behavior	244	4.86 ± 0.38	5.00 [5.00, 5.00]	3–5	238	4.90 ± 0.30	5.00 [5.00, 5.00]	4–5
Health outcomes	244	4.89 ± 0.33	5.00 [5.00, 5.00]	3–5	238	4.92 ± 0.26	5.00 [5.00, 5.00]	4–5
Government support	244	3.71 ± 0.85	4.00 [3.00, 4.00]	2–5	238	3.62 ± 0.93	4.00 [3.00, 4.00]	1–5
Information avoidance	244	4.32 ± 0.67	4.00 [4.00, 5.00]	2–5	238	4.37 ± 0.62	4.00 [4.00, 5.00]	3–5

Source: author’s contribution.

### 3.4 Indicator framework

**Figure 2:**
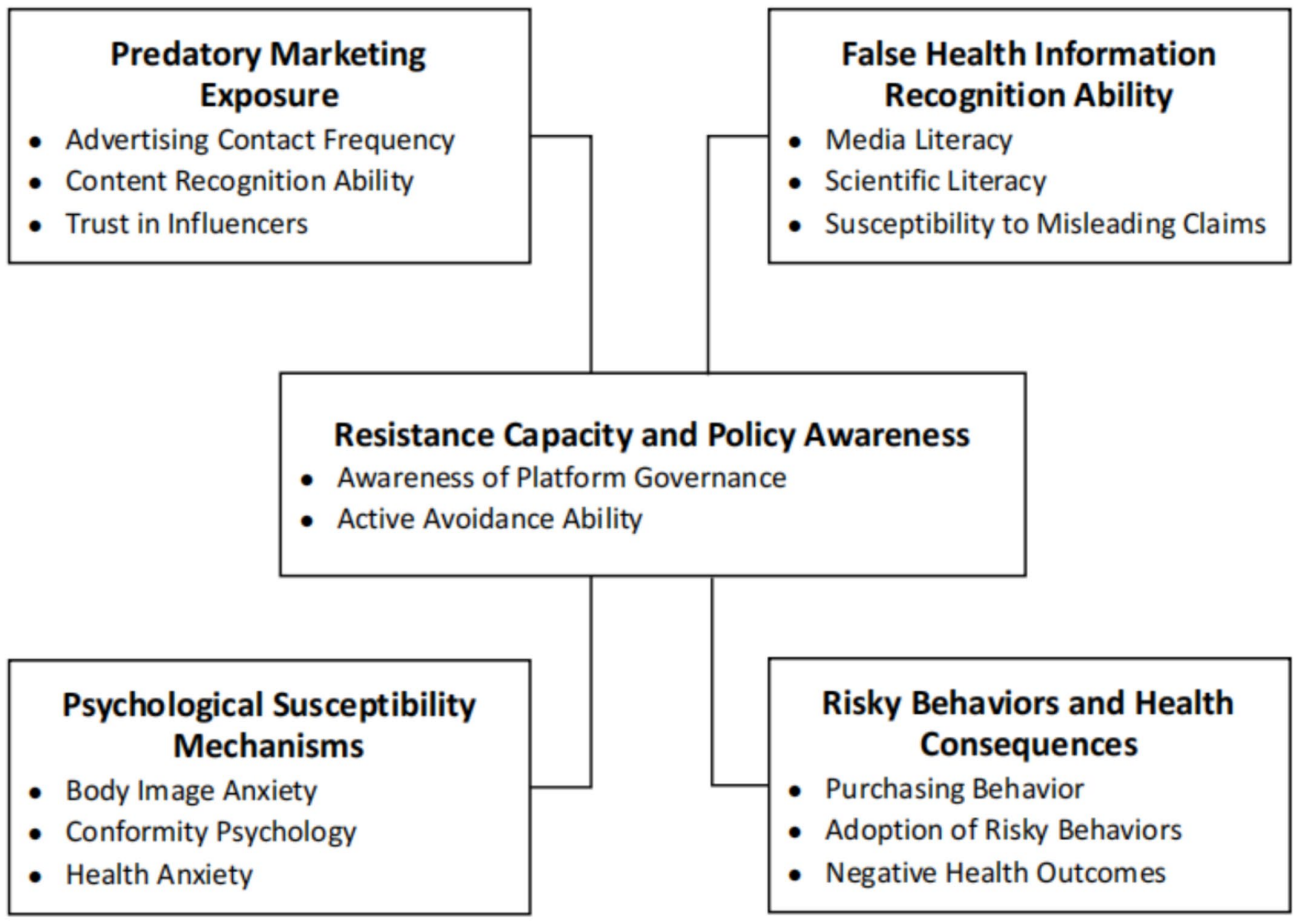
Conceptual framework of risky health marketing exposure and its psychosocial Mechanisms. Source: author’s contribution.

Based on a comprehensive review of prior research and theoretical integration, this study develops a structured, five-dimensional indicator framework to assess individual responses to predatory health marketing on digital platforms. The first dimension, Predatory Marketing Exposure, includes advertising contact frequency, content recognition ability, and trust in influencers, capturing the extent to which individuals are exposed to and aware of marketing disguised as personal health advice. The second dimension, False Health Information Recognition Ability, reflects individuals’ capacity to distinguish credible from misleading content through media literacy, scientific literacy, and susceptibility to persuasive but inaccurate claims. The third dimension, Psychological Susceptibility Mechanisms, incorporates body image anxiety, conformity tendencies, and health-related anxiety, emphasizing emotional and cognitive vulnerability to marketing narratives. The fourth dimension, Risky Behaviors and Health Consequences, addresses behavioral outcomes such as purchasing decisions, adoption of risky health practices, and resulting negative health effects. The fifth dimension, Resistance Capacity and Policy Awareness, focuses on awareness of platform governance and the ability to actively avoid or report misleading content. This framework offers a standardized basis for empirical measurement and is applicable to structural equation modeling or pathway analysis in health communication research. In this study, media literacy is understood as the ability to access, analyze, evaluate, and create media content in ways that recognize persuasive and potentially misleading techniques, whereas scientific literacy refers to the capacity to understand, evaluate, and appropriately trust scientific evidence and expert consensus in health-related contexts (11, 44) (see Figure 2).

## 4 Model specification

This chapter presents and assesses the model that links exposure to predatory health marketing with psychological susceptibility, belief in misinformation, risky health behaviors, and health outcomes. The first part reports univariate descriptive statistics, distributional properties, and sampling adequacy tests to show that the data are suitable for factor and structural modeling. The second part evaluates the measurement model through confirmatory factor analysis, examining factor loadings, reliability, and convergent and discriminant validity. The third part estimates the structural paths and tests the main hypotheses about how exposure, susceptibility, false beliefs, risky behaviors, and outcomes are connected. The fourth part focuses on mediation analysis, tracing how misinformation influences health outcomes through sequential psychological and cognitive processes. The fifth part investigates whether Government Support and Information Avoidance moderate key links in the mechanism. The final part explores subgroup differences and conducts robustness checks, comparing the proposed model with an alternative specification in order to demonstrate the central role of false beliefs.

### 4.1 Descriptive statistics and normality testing

The purpose of this section is to provide an overview of the distributional characteristics of the study variables and to assess their suitability for subsequent factor and structural modeling. Before estimating the measurement and structural models, it is essential to examine the central tendencies, variability, and normality indicators of the main constructs so that the data foundation for later analyses is clearly established.

Table 4 presents the descriptive statistics and normality indicators for the study variables. The means ranged from 3.08 to 4.91, showing that participants reported generally moderate to high levels of exposure to predatory health marketing, psychological susceptibility, belief in false health claims, risky health behaviors, and perceived negative health outcomes. Standard deviations were relatively small (0.30 to 1.44), indicating moderate variation without substantial dispersion. With regard to distributional features, all variables displayed negative skewness (−0.09 to −3.02) and kurtosis values between −1.33 and 8.09. Although several kurtosis values exceed the ±1 reference range, such patterns are frequently observed in research on attitudes and health-related behaviors and remain acceptable for maximum likelihood–based analyses, particularly when sample size is adequate. To further assess data suitability for factor analysis, the Kaiser–Meyer–Olkin (KMO) statistic and Bartlett’s test of sphericity were conducted. The KMO value of 0.913 indicates strong sampling adequacy, and the significant Bartlett result (χ^2^ = 2184.7, *p* < 0.001) confirms that the correlation matrix is appropriate for factor extraction. Overall, these indices show that the data meet the basic requirements for confirmatory factor analysis (CFA) and structural equation modeling (SEM), supporting their use in subsequent measurement and structural evaluations.

**Table 4:** Descriptive statistics and normality test results.

Variable	M	SD	Skewness	Kurtosis
Exposure to predatory marketing	3.08	1.44	−0.09	−1.33
Psychological susceptibility	4.20	0.88	−0.68	−0.69
Belief in false health claims	4.72	0.52	−1.65	1.83
Risky health behaviors	4.88	0.34	−2.77	7.14
Negative health outcomes	4.91	0.30	−3.02	8.09

Source: author’s contribution.

### 4.2 Measurement model (CFA)

This section evaluates the measurement quality of the latent constructs prior to testing the structural relationships. Establishing a sound measurement model is essential for ensuring that each construct is represented accurately and that the observed indicators capture the intended theoretical dimensions. The analysis examines factor loadings, composite reliability, convergent validity, and discriminant validity to verify that the items are internally consistent, conceptually coherent, and empirically distinct from one another.

The measurement properties of the constructs were examined using confirmatory factor analysis. As shown in Table 5, all standardized loadings fall within acceptable ranges, with values between 0.74 and 0.86 for Exposure, 0.72 and 0.84 for Psychological Susceptibility, 0.73 and 0.88 for Belief in Misinformation, 0.71 and 0.83 for Risky Behaviors, and 0.72 and 0.84 for Negative Health Outcomes. These loading patterns indicate that each set of items provides a consistent representation of its underlying construct. Composite reliability values range from 0.85 to 0.88, reflecting adequate internal consistency. Convergent validity is supported by AVE values between 0.58 and 0.63, all exceeding the recommended minimum. Discriminant validity is further indicated by the HTMT values, which fall between 0.72 and 0.79 and remain below established thresholds. Overall, the measurement model demonstrates stable reliability and validity, providing a sound basis for the subsequent structural analysis.

**Table 5:** Reliability and validity results of the measurement model.

Construct	Standardized loadings (range)	CR	AVE	HTMT (Max)
Exposure	0.74–0.86***	0.87	0.62	0.72
Psychological susceptibility	0.72–0.84***	0.86	0.59	0.76
Belief in misinformation	0.73–0.88***	0.88	0.63	0.79
Risky behaviors	0.71–0.83***	0.85	0.58	0.74
Negative health outcomes	0.72–0.84***	0.86	0.6	0.77

Source: author’s contribution. *p < 0.05; **p < 0.01; ***p < 0.001.

### 4.3 Structural model estimation (SEM)

To further examine the core relationships proposed in the structural model, a series of path analyses were conducted, and the results are summarized in Table 6. This table reports the standardized path coefficients, standard errors, confidence intervals, and significance levels for each hypothesized relationship, enabling a clear assessment of how exposure, psychological susceptibility, false beliefs, and risky behaviors are linked within the model and how they jointly contribute to negative health outcomes.

**Table 6:** Results of structural path estimates.

Path	β	SE	95% CI	*p*-value
Exposure → psychological susceptibility	0.775***	0.017	[0.742, 0.809]	***
Psychological susceptibility → belief in misinformation	0.565***	0.035	[0.496, 0.633]	***
Belief in misinformation → risky behaviors	0.484***	0.052	[0.382, 0.586]	***
Risky behaviors → negative health outcomes	0.356***	0.067	[0.225, 0.487]	***

Source: author’s contribution. *p < 0.05; **p < 0.01; ***p < 0.001.

The structural paths in Table 6 demonstrate a continuous psychological, cognitive, and behavioral process through which misleading health information affects individual judgment and health outcomes. Media exposure showed a strong positive association with psychological susceptibility (*β* = 0.775***), indicating that repeated contact with emotionally charged, sensational, or superficially scientific content increases uncertainty and reduces the capacity for careful evaluation. In such situations, individuals tend to rely more on intuitive judgments, which makes misleading information appear plausible. Elevated susceptibility then contributed to the acceptance of false health beliefs (*β* = 0.565***). This pattern reflects the persuasive force of misinformation, which often appeals to emotion, creates an impression of expertise, or constructs compelling narratives that are taken as credible in the absence of systematic verification. Once these beliefs become internalized, they translated into a higher likelihood of engaging in risky health behaviors (*β* = 0.484***), suggesting that distorted cognition can readily shape health-related decisions, including adherence to unverified practices or neglect of professional guidance. These behavioral choices ultimately resulted in more negative health outcomes (β = 0.356***), showing that cognitive deviations have tangible effects on physical and psychological well-being. Although the magnitude of effects gradually decreased along the pathway, each link remained statistically robust, suggesting that the influence of misleading information accumulates through successive psychological and behavioral stages rather than appearing abruptly. At the same time, the model explained less than half of the variance in health outcomes, implying the presence of other influential factors, such as media literacy, critical evaluation skills, peer or social norms, and platform-level mechanisms. These results provide theoretical insight into how misinformation operates across multiple layers of psychological processing and highlight the practical need to strengthen public judgment capacity, improve information environments.

### 4.4 Mediation effects (bootstrap, 5,000 samples)

To further clarify the internal mechanisms through which misinformation exerts its influence, a series of mediation analyses were conducted, and the results are summarized in Table 7. This table reports the indirect effect estimates obtained through bootstrap procedures, allowing a direct assessment of how exposure to misinformation is transmitted through psychological susceptibility, belief formation, and risky behaviors before ultimately shaping health outcomes.

**Table 7:** Mediation effect analysis.

Path	Indirect effect (β)	95% CI	*p*-value
Exposure → psychological susceptibility → belief in misinformation	0.438***	[0.129, 0.187]	***
Exposure → psychological susceptibility → belief in misinformation → risky behaviors	0.212***	[0.034, 0.069]	***
Full chain: exposure → … → negative health outcomes	0.075***	[0.008, 0.025]	***

Source: author’s contribution. *p < 0.05; **p < 0.01; ***p < 0.001.

By presenting effect sizes alongside confidence intervals, Table 7 provides a comprehensive overview of the sequential pathways that link cognitive and psychological processes to behavioral and physiological consequences. As shown in Table 7, all mediation paths were statistically significant, with 95% confidence intervals excluding zero. Exposure increased psychological susceptibility, which in turn strengthened misinformation beliefs (*β* = 0.438***). The extended mediation chain from psychological susceptibility to beliefs and then to risky behaviors also showed a significant indirect effect (*β* = 0.212***). The full pathway—from exposure through psychological and cognitive mechanisms to negative health outcomes—remained significant as well (*β* = 0.075***), indicating a multi-stage transmission process. Although effect sizes were moderate, the results underscore that the influence of misinformation accumulates across psychological, cognitive, and behavioral layers, highlighting the importance of interventions that address these mechanisms simultaneously rather than focusing on a single stage.

### 4.5 Moderation effects

To further analyze whether individual defensive mechanisms shape the psychological and behavioral effects of misinformation, a moderation analysis was conducted, and the results are presented in Table 8. This table summarizes the interaction effects estimated using OLS, allowing an examination of how Government Support and Information Avoidance alter the strength of key pathways within the misinformation process.

**Table 8:** Results of moderation effect analysis.

Moderator	Path	β	SE	t-value	*p*-value
Government support	Exposure × governance support → psychological susceptibility	−0.055	0.035	−1.783	*
Information avoidance	Belief × information avoidance → Risky behaviors	−0.177	0.063	−2.171	**

Source: author’s contribution. *p < 0.1; **p < 0.01; ***p < 0.001.

**Figure 3:**
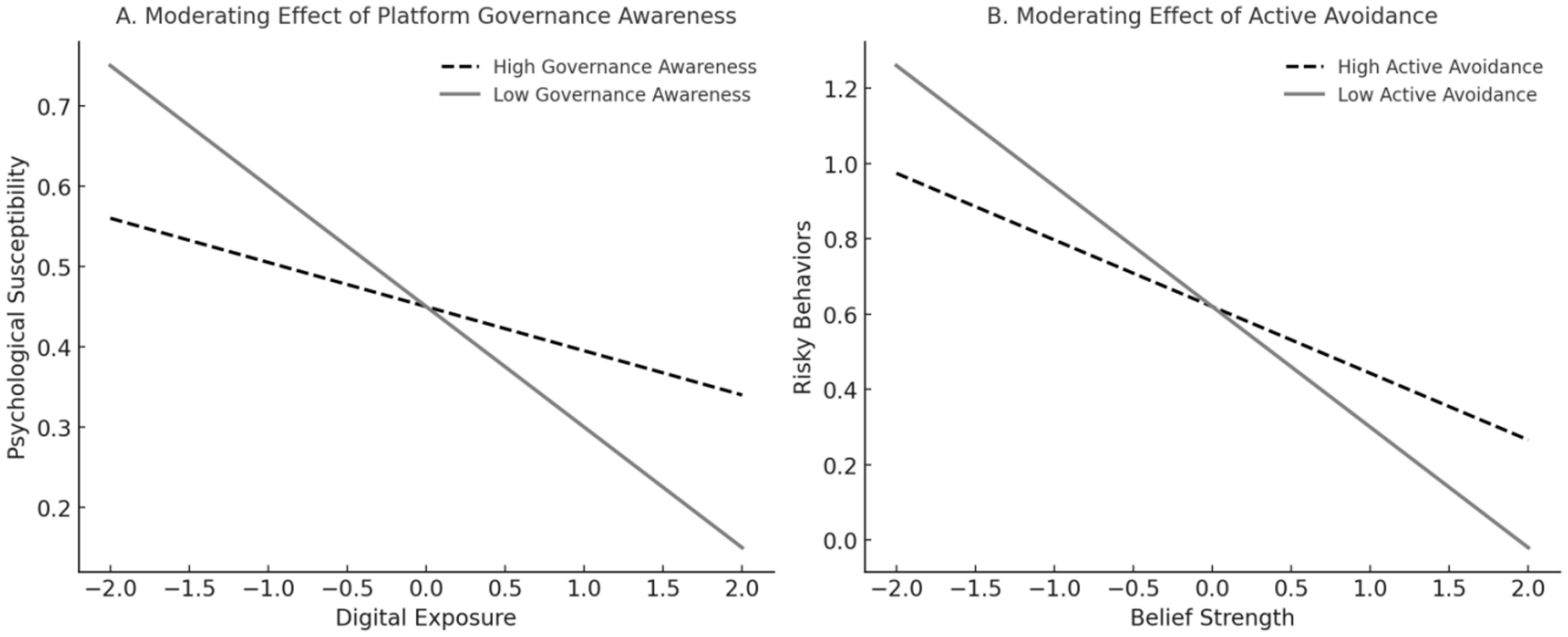
Simple slopes of moderation effects. **(A)** Moderating effect of platform governance awareness on the relationship between digital exposure and psychological susceptibility. **(B)** Moderating effect of active avoidance on the relationship between belief strength and risky behaviors. Source: author’s contribution.

As shown in Table 8 and Figure 3, both moderators exerted significant negative influences on the misinformation pathway. Government Support weakened the effect of exposure on psychological susceptibility (*β* = −0.055, t = −1.783, *p* < 0.05), indicating that individuals who are more aware of governance measures—such as content regulation and verification cues—are less vulnerable even under high exposure. Information Avoidance also reduced the translation of false beliefs into risky behaviors (β = −0.177, t = −2.171, *p* < 0.01), suggesting that behaviors such as blocking and ignoring false content serve as effective behavioral defenses. Although these moderating effects were modest in size, they highlight the need for combined efforts across platform governance, technological filtering, and user behavior to effectively mitigate the influence of misinformation in digital environment.

### 4.6 Multi-group analysis (MGA)

To further examine whether the structural relationships in the model vary across different population segments, a series of multi-group analyses were conducted, and the results are summarized in Table 9. This table presents the subgroup-specific path coefficients estimated using OLS, allowing a direct comparison of how misinformation exposure and behavioral mechanisms operate across gender, social media usage frequency, and age categories.

**Table 9:** Results of multi-group analysis (OLS).

Grouping variable	Path comparison	β₁	β₂
Gender	Belief in misinformation → risky behaviors	Male = 0.370***	Female = 0.676***
Usage frequency	Exposure → psychological susceptibility	High frequency = 0.807***	Low frequency = 0.881***
Age group	Risky behaviors → negative health outcomes	Youth = 0.398***	Middle-aged/Older = 0.194***

Source: author’s contribution. *p < 0.05; **p < 0.01; ***p < 0.001.

As shown in Table 9, for gender, the effect of false beliefs on risky behaviors was substantially stronger among women (*β* = 0.676***) than men (*β* = 0.370***), suggesting that female users may be more behaviorally influenced once misinformation is internalized. Regarding usage frequency, exposure to misinformation significantly increased psychological susceptibility in both groups, with strong effects among high-frequency users (*β* = 0.807***) and low-frequency users (*β* = 0.881***), indicating that the psychological impact of misinformation is robust regardless of intensity of social media use. For age, risky behaviors had a greater impact on negative health outcomes among younger individuals (*β* = 0.398***) compared with middle-aged and older adults (*β* = 0.194***), implying heightened vulnerability among youth when risky actions escalate into adverse consequences. Overall, these results demonstrate meaningful heterogeneity across demographic subgroups and underscore the importance of tailoring health communication and intervention strategies to differences in gender, exposure patterns, and age-related susceptibility.

### 4.7 Robustness checks

As shown in Table 10, robustness checks were conducted by comparing the original structural equation model with an alternative model that removed the false belief construct. The original model demonstrated excellent model fit across all indices (χ^2^/df = 0.653, CFI = 1.000, TLI = 1.004, RMSEA = 0.000, SRMR = 0.021), all of which far exceed the conventional thresholds for good fit. The information criteria were also lower (AIC = 1709, BIC = 1743), indicating a highly parsimonious model with strong explanatory ability.

**Table 10:** Results of robustness checks.

Model	χ^2^/df	CFI	TLI	RMSEA	SRMR	AIC	BIC	Remarks
Original Model (with False Beliefs)	0.653	1.000	1.004	0.000	0.021	1709	1743	0.653
Alternative Model (without False Beliefs)	21.140	0.877	0.753	0.204	0.096	1813	1851	21.140

Source: author’s contribution.

When the false belief construct was removed, the overall model fit deteriorated substantially. The χ^2^/df ratio rose sharply to 21.140, CFI dropped to 0.877, and TLI fell to 0.753, suggesting weakened incremental and comparative fit. RMSEA increased markedly to 0.204, and SRMR rose to 0.096, both indicating considerable misfit. In addition, AIC and BIC increased to 1813 and 1851, respectively, further signaling a reduction in model adequacy and parsimony. Taken together, these results clearly demonstrate that the false belief construct plays a central and indispensable role in the model. Removing this variable not only weakens specific structural paths but also disrupts the overall coherence and stability of the model. The robustness checks therefore provide strong empirical support for the theoretical framework and confirm the necessity of including false beliefs as a core explanatory mechanism.

## 5 Discussion

This chapter discusses the main findings in light of existing work on health misinformation, predatory marketing, and platform governance, and considers what they mean for theory and practice. The first part reflects on the limited yet meaningful buffering role of Government Support and Information Avoidance, relating these results to broader debates about individual level interventions and structural amplification on digital platforms. The second part analyzes differences in the mechanism across gender, age, and usage patterns, and explains how the inclusion of measurement invariance testing strengthens the interpretation of these group patterns. The third part focuses on the mediating role of false beliefs and questions the reliance on exposure based indicators as the main benchmark for governance performance, arguing for a stronger focus on belief formation. Together, these discussions connect the empirical results to current theoretical conversations, draw out implications for policy makers, platforms, health professionals, and educators, and prepare the ground for the limitations and future research directions outlined in the conclusion.

### 5.1 Limited individual-level effects and structural dilution at the platform level

The findings of this study indicate that Government Support and Information Avoidance provide a meaningful but limited buffering effect against misleading health content. This outcome is broadly consistent with previous research showing that media literacy, prebunking, and inoculation interventions can enhance resistance to misinformation (7, 10, 20). Yet much of this evidence is derived from controlled environments in which exposure conditions, emotional cues, and cognitive load are tightly regulated, producing stronger intervention effects than those typically observed in naturalistic digital settings (8, 45). By contrast, the present study, based on real-world platform dynamics, demonstrates that attention-driven recommendation systems amplify emotionally arousing and sensational content (15, 23), creating a structural amplification mechanism that weakens the protective influence of individual-level strategies. This interpretation aligns with arguments that the effectiveness of cognitive interventions is constrained by the broader information environment and that misinformation reflects systemic dynamics rather than solely individual deficits (6, 24). This pattern echoes long-standing insights from risk communication, which emphasize that effective interventions must address both cognitive appraisal and affective responses rather than relying on purely informational correction (34, 46–48). Recent discussions in the governance literature similarly emphasize structural responsibility, calling for greater accountability, transparency, and oversight at the platform level. Against this backdrop, the findings of this study illustrate that user vigilance alone cannot counteract the algorithmic forces that systematically amplify misleading content.

### 5.2 Group differences in mechanism pathways and the stability of measurement structure

The multi-group analysis revealed notable differences in the strength of structural paths across gender, age, and usage frequency. Women exhibited greater sensitivity in the transition from false beliefs to risky behaviors, whereas younger and high-frequency users showed stronger responses in the early stage linking exposure to psychological vulnerability. These patterns are consistent with previous evidence demonstrating that adolescents, heavy social media users, and individuals with heightened affective reactivity are more susceptible to health-related misinformation, and they parallel findings in domains such as fitness culture, body image content, and supplement marketing, where similarly vulnerable subgroups have been documented (3, 4, 11). Unlike studies that rely solely on direct comparisons of means or path coefficients, the present study incorporated measurement invariance testing to ensure equivalence in structural configuration, factor loadings, and selected intercepts across groups. This approach mitigates the risk of drawing conclusions based on artificial differences caused by measurement nonequivalence, thereby strengthening the validity of the observed heterogeneity and addressing a methodological gap that has been frequently overlooked in earlier research (21, 49). These findings also resonate with scholarship on echo chambers and homophily, which suggests that differences in platform interaction rhythms, emotional trigger thresholds, and content recommendation patterns can shape users’ psychological vulnerability at different stages of a mechanism, causing the same measurement items to function differently across user types and usage contexts (50, 51). Generally, the evidence indicates that the influence of misleading health information cannot be understood as a uniform linear process operating similarly across all demographic groups. Interpretations and generalizations of misinformation effects should therefore avoid assuming homogeneity. Crucially, the present study contributes an analytical framework capable of distinguishing stable elements of the mechanism from components that shift according to demographic characteristics and media use patterns. This dual perspective moves the field beyond interpreting effects through population averages and instead highlights the conditional and differentiated nature of susceptibility and behavioral outcomes. Such an integrated approach not only provides a theoretical basis for explaining inconsistencies across prior studies but also offers more robust guidance for developing targeted prevention strategies, identifying high-risk subgroups, and designing stratified interventions.

### 5.3 The mediating role of false beliefs and the limits of exposure-based KPIs

Robustness analyses in this study indicate that false beliefs occupy a central position in the mechanism through which predatory health marketing shapes individual judgments and behaviors. When the belief construct was removed, model fit deteriorated sharply, structural paths were disrupted, and explanatory power declined, demonstrating that risky behaviors do not arise immediately from isolated exposure but rather emerge from a gradual process in which distorted claims are repeatedly reinforced, internalized, and integrated into one’s cognitive framework. This mechanism aligns with dual-process theories and findings on familiarity-based truth perceptions and belief consolidation, which show that repeated exposure, processing fluency, and affectively charged cues encourage intuitive judgments and make erroneous beliefs highly stable and resistant to correction once formed (9, 12, 45). In contrast to these deeper psychological dynamics, prevailing platform governance continues to rely primarily on exposure-reduction indicators—such as content removal, demotion, or impression suppression—to evaluate performance. Although these measures can temporarily reduce the visibility of harmful content, they seldom reach the underlying cognitive structures users have already developed, leaving distorted beliefs capable of resurfacing through memory retrieval, social reinforcement, or later re-exposure (19, 26). Prior evidence further suggests that warning labels and corrective notices often reduce engagement but exert limited influence on belief revision, creating an illusion of improved governance while the core mechanism remains largely unaddressed (37, 38). The findings of this study reinforce the need to intervene at the level of belief formation rather than relying solely on suppressing surface-level exposure. By combining a sequential pathway model with rigorous robustness checks, this study demonstrates the pivotal role of false beliefs across the full chain linking exposure, psychological susceptibility, cognitive processing, behavioral choices, and health outcomes. Taken together, these results show that effective governance must restrict the emergence, reinforcement, and diffusion of erroneous claims through institutional and structural measures, supporting a shift from visibility-based control toward upstream interventions that weaken the cognitive foundations upon which risky behaviors are built.

## 6 Conclusion

This study constructs and validates a sequential pathway model that links exposure, psychological vulnerability, false beliefs, and behavioral deviation in order to examine the mechanisms through which health misinformation and covert promotional practices on social media influence individual cognition and behavior. To address the research questions posed in Chapter 1, the empirical evidence provides clear answers. Regarding RQ1, repeated and emotionally stimulating exposure to misleading health content significantly increases psychological vulnerability and biases individuals’ subjective risk judgments. Regarding RQ2, media literacy and scientific literacy offer only limited improvements in the recognition of false information because their protective effects are often constrained by algorithmic amplification and emotionally engineered content. Regarding RQ3, psychological vulnerability and false beliefs jointly constitute the central mediating mechanisms through which exposure affects health-related behaviors, indicating that behavioral deviation arises from a continuous cognitive and emotional processing chain rather than isolated encounters with misinformation. Regarding RQ4, sustained exposure produces measurable negative outcomes, including stronger endorsement of false beliefs, greater likelihood of engaging in risky health behaviors, and the accumulation of adverse health consequences, which confirms the cumulative and path-dependent nature of these effects. The study further shows that repeated exposure to misleading content heightens psychological vulnerability and facilitates the formation and consolidation of false beliefs. These beliefs function as the primary mechanism through which behavioral changes occur and through which negative health outcomes accumulate. Although awareness of governance and information avoidance provide modest buffering effects, their influence is considerably restricted by platform architectures that prioritize emotionally charged and sensational material. The multi-group analyses indicate differentiated sensitivities across demographic groups. Women display stronger transitions from belief to behavior, while younger individuals and high-frequency users show heightened psychological and behavioral reactivity to exposure. Robustness checks highlight the essential role of false beliefs in the explanatory chain. When this construct is removed, the coherence and interpretive strength of the model decline sharply, demonstrating that behavioral deviation is the outcome of sustained cognitive distortion rather than incidental exposure.

Building on these findings, the study reconceptualizes predatory health content as a strategically engineered form of digital health communication that incorporates commercial incentives, pseudo-scientific claims, and emotionally charged narratives within personalized platform environments. It operates by concealing promotional intent, by using algorithmic amplification to enhance visibility, and by drawing on socially reinforced credibility cues to shape individual beliefs and behaviors. In comparison with ordinary misleading information, predatory health content is characterized by its commercial logic, its relational narrative design, and its systematic use of influencer trust, aesthetic appeal, and interactive affordances in shaping decisions. This refined conceptualization clarifies the structural and psychological characteristics that distinguish predatory content from general misinformation and strengthens the theoretical contribution of this research by linking micro-level belief formation processes with platform-level architectures. Overall, the evidence suggests that the influence of digital health misinformation does not arise solely from deficits in media literacy. Instead, it emerges from the interaction of individual characteristics, cognitive and emotional processes, and structural features of platform environments. This dynamic helps explain why literacy-based interventions often fail to produce lasting effects in real-world settings. The integrated framework developed in this study addresses long-standing gaps in the literature by providing a coherent explanatory chain that connects exposure, vulnerability, belief formation, and behavioral outcomes, while also showing how platform structures may limit the effectiveness of individual-level interventions. The identification of group-specific variations and the stability of the underlying mechanisms offers a foundation for designing more targeted governance strategies. Although this study is limited by its cross-sectional design and its focus on a single national context, it nevertheless provides a systematic and contextually informed perspective for understanding the spread of digital health misinformation, identifying key points for intervention, and informing governance strategies that incorporate both structural and psychological considerations.

### 6.1 Limitation

This study provides important insights into how exposure to predatory health marketing on social media affects individual cognition and behavior within the Chinese digital context. However, the extent to which the findings can be generalized internationally remains limited. Key constructs such as platform governance, psychological vulnerability, and media trust are deeply embedded in China’s unique sociopolitical environment, where platform regulation, state intervention, and user expectations differ substantially from those in other countries. These contextual differences may influence the pathways identified in this model, particularly in societies with more decentralized media systems or varying levels of digital surveillance. Moreover, health-related beliefs and responses to manipulative content are often shaped by cultural norms, institutional trust, and information ecosystems, all of which vary across regions. Given that the data were collected from a single national context, future research should adopt cross-cultural comparative designs, localize measurement instruments, and examine how socio-digital infrastructures condition individual responses to health misinformation. Such efforts are essential to advancing a more globally valid and culturally sensitive understanding of digital health risks.

### 6.2 Recommendation

The findings of this study show that misleading health content on social media does not influence users through a single moment of exposure but through a gradual process in which repeated contact heightens psychological vulnerability, strengthens inaccurate beliefs, and ultimately shapes risky behaviors that harm health outcomes. This progression indicates that governance efforts limited to regulating content visibility are insufficient, because they overlook the deeper cognitive and emotional mechanisms that allow false beliefs to develop and become resistant to correction. In domains such as supplement promotion, extreme dieting, and high-risk fitness routines, persuasive narratives, pseudo-scientific claims, and influencer endorsement often create a sense of credibility that encourages intuitive rather than analytical judgment, making individuals more susceptible to internalizing misinformation. Strengthening evidence-based review procedures, enhancing the verification of health-related claims, and enforcing stricter disclosure requirements for influencers can help reduce the spread of misleading narratives and limit their influence on belief formation. The multi-group results further reveal that younger users, those who use social media frequently, and individuals with higher psychological vulnerability are more sensitive to each stage of this belief formation pathway, underscoring the need for interventions that are tailored to specific high-risk groups rather than broad, undifferentiated public health messaging. Public health agencies may therefore consider developing targeted early interventions, such as prebunking strategies for heavy users, cognitive regulation training for individuals with strong emotional reactivity, and digital health literacy programs that equip users with basic evaluative skills before they encounter online health content. Although perceptions of platform governance and Information Avoidance demonstrated some buffering effects, these forms of self-protection remain insufficient to counteract the systemic biases generated by algorithmic recommendation systems. A more effective governance framework would shift from passive filtering to proactive guidance by incorporating credibility labels, authoritative prompts, and evidence-based recommendation mechanisms that reduce reliance on intuitive processing and weaken the psychological conditions that enable false beliefs to take hold. Future research could employ longitudinal designs to trace the process through which beliefs emerge, solidify, and influence behavior, while experimental studies could further clarify how emotional arousal, cognitive load, and algorithmic environments interact to shape susceptibility. Comparative analyses across cultural contexts and platform ecosystems may help determine whether the mechanisms observed in this study generalize beyond the current setting, and network-level investigations into user clustering, dissemination pathways, and influencer ecosystems could enrich theoretical understanding of how misinformation spreads and offer evidence to support earlier, more precise, and more systemic governance interventions.

## Data Availability

The raw data supporting the conclusions of this article will be made available by the authors, without undue reservation.

## Footnotes

1
www.wjx.cn
