# Supplementary material for: Predatory marketing and false health promotion on social media: risk pathways in diet, fitness, and supplement communication
Source: Front Public Health. 2026 Feb 23;14:1709812. doi: 10.3389/fpubh.2026.1709812 (PMC12968223; doi:10.3389/fpubh.2026.1709812)
Supplement: Supplementary file 1 [file Image_1.pdf]

SUPPLEMENTARY STATEMENT

This email serves to supplement the Research Ethical Statements for the manuscript "Predatory Marketing and False Health Promotion on Social Media: Risk Pathways in Diet, Fitness, and Supplement Communication", the manuscript ID is 1709812.

This manuscript has been reviewed for ethical compliance, and the following supplementary statement outlines the ethical considerations adhered to during the conduct of the study:

The social media data was accessed and analyzed in accordance with the Dou Yin(Chinese TikTok) platform's terms of use and all relevant institutional/national regulations.

Reviewing Institution (Seal): 赣州理工职业学院 科研产业处

Approval Date: 26/9/2025
